# Early identification of at-risk children: service improvement study using immunisation status

**DOI:** 10.3399/BJGPO.2022.0035

**Published:** 2023-01-11

**Authors:** Rachael Gail Kilner, Adam Glen Cunliffe, Carla Stanke

**Affiliations:** 1 Herne Hill Road Medical Practice, London, UK; 2 Lambeth Early Action Partnership (LEAP), London, UK

**Keywords:** general practice, health visiting, child health, risk factors, adverse childhood experiences, immunisation

## Abstract

**Background:**

Children who have adverse childhood experiences (ACEs) tend to have more physical and mental health problems when they are adults compared with people who do not have ACEs. Evidence suggests that partial or no immunisation status can be associated with factors (including ACEs) that make children at higher risk of poor outcomes than immunised children.

**Aim:**

To explore the idea that ‘missed immunisations’ could be used as a proxy indicator in identifying children at risk of worse outcomes.

**Design & setting:**

Service improvement study in seven GP practices in south London, UK.

**Method:**

Children aged 0–3 years who were ≥3 months late for immunisations were identified; their computer notes were reviewed during interdisciplinary meetings between health visitors (HVs) and GP practice staff. A bespoke template was used to guide discussions and to record action plans. Evaluation methods included a survey of practitioners and anonymised questionnaires about care management for a sample of children.

**Results:**

Issues of concern, including some ACEs (for example, domestic abuse, mental health concerns in parent), were identified in 57% of children. Ninety-four per cent of practitioners found multidisciplinary meetings useful; 62% of practitioners changed the way they thought about providing care to very young children and their families. Of the children discussed during multidisciplinary meetings, 38% subsequently caught up on immunisations.

**Conclusion:**

‘Late for immunisations’ appears to be a useful indicator for proactively identifying children with issues that make them at risk of poorer outcomes. Integrated working between GPs and HVs is important for ensuring targeted care is provided to families.

## How this fits in

Identification of ACEs from children’s GP computer notes can be challenging owing to incomplete information entered and coding issues. ‘Late for immunisations’ appears to be a useful indicator for identifying children who may have experienced ACEs and is easy to search for. Targeting these children for an in-depth review of their notes and an interdisciplinary discussion between GPs and HVs could help direct limited resources to families who might benefit most.

## Introduction

A child’s health is largely influenced by the broader environment in which they live; these influences include the health behaviours of the parents, a family’s ability to provide care, and the wider socioeconomic conditions of the family.^1^ A growing body of research has identified that children who have ACEs have more physical and mental health problems as adults compared with people who do not have ACEs.^2,3^ It makes sense for child health systems and providers of care to work together when any of the wider determinants of health have a detrimental effect on a child’s wellbeing. Identifying children affected by ACEs and other social issues, and maximising local systems and preventive strategies, may help reduce the incidence of associated health problems and avoid escalation to more costly acute services.

General practice has good potential to initiate more proactive engagement with families to improve the health and wellbeing of local populations,^4^ but there is currently no systematic way for healthcare providers to proactively identify children at increased risk of worse outcomes owing to social circumstances. Children in Lambeth are often only identified and put on a GP practice’s child protection list once children’s social services are involved or if domestic violence is known to be present in the family. Clinicians often use professional judgement to identify children with social and environmental factors earlier, but this is not always standardised across practices.

An algorithm that would search for children with ACEs, as described in the literature,^2,3^ initially seemed a good option, but in practice this was difficult to achieve owing to some risk factors being documented in parents’ notes rather than children’s notes. For example, domestic abuse or drug or alcohol abuse in family member and other risk factors — such as parent in prison or parental separation — are often not coded within GP computer notes. In addition, some information is held by different databases, which is not always accessible by GPs; for example, HV, social services, or criminal records data.

A further limitation of using ACE factors is that one of the most important variables for children at risk of poor outcomes — poverty — is not included in the initial list of ACEs, is difficult for a healthcare practitioner to quantify, and is not coded within GP notes.

Given the logistical challenges in searching for children with specific social risk factors, the authors considered whether one variable could be used as a proxy indicator to identify these children. Evidence suggests that partial or no immunisation status can be associated with factors that make children at higher risk of poor outcomes.^5–7^ Other indicators considered included accident and emergency (A&E) repeated attendances, or missed hospital outpatient appointments. It was concluded that different electronic coding practices between GP practices would result in too many conflicting variables to use either of these as the proxy indicator. Therefore, the GP Connect project was developed to explore the idea that missed immunisations could be used as a proxy indicator in identifying children in Lambeth at risk of worse outcomes.

## Method

As part of the Lambeth Early Action Partnership (LEAP) work to improve early years outcomes in specific areas of the borough, 17 GP practices located within four Lambeth wards — Stockwell, Vassall, Coldharbour, and Tulse Hill — were visited by the LEAP GP and public health specialist. Their aims were to promote awareness of local children’s services, discuss proposals for the GP Connect project, and to identify whether GP and HV meetings were occurring. An invitation to participate in GP Connect was subsequently sent to all practices.

Practices ran a computer search to identify children aged 0–3 years, registered in the four wards using postcodes, who were ≥3 months late for any immunisations.^8^ While there is no standard definition for delayed immunisation, some studies use 28 days as the cut off for ‘late for immunisation’.^9^ It was felt this was too short a time to allow for exclusion of short-term barriers to getting children immunised; for example, availability of appointments and holidays.

Practices were asked to arrange 4–6 weekly meetings between the GP and HV if these were not already scheduled as part of Lambeth Safeguarding Children Partnership guidance,^10^ and the wider practice team was also encouraged to attend. The GP Connect meetings did not differ in format from previously arranged GP and HV meetings; the only addition was the discussion of children who were late for immunisations beyond those already on the practice safeguarding register.

The pilot phase ran from April–November 2019 and at least four GP and HV meetings per practice were required during this time. Owing to time constraints, it was decided that five children from the computer search list were to be discussed per meeting, yielding a minimum of 20 children discussed per practice.

A bespoke template was developed to be completed during the GP and HV meetings and to help guide the discussion (Supplementary Figure S1).

Information was collected from GP and HV notes and included any social services information recorded on these systems; Lambeth GPs do not currently have direct access to social services computer records. This information included the following:

- Any missed outpatient appointments and/or if the child had attended A&E more than twice in the past 12 months;
- If the child is on the child protection register and under which category;
- If a child is recorded as ‘looked after’ or as a ‘child in need’;
- Family history of mental illness, learning disability, drug or alcohol abuse, domestic abuse, or if family member is on child protection register;
- Social history: if the family is at risk of social isolation, has poor family relationships, receives benefits, or rents their house from the council.

Practices were asked to develop an action plan for each child discussed. Possible actions included the following: referral to children’s centre, health visitor, GP, or hospital paediatric services; drug or alcohol service referral for family member; and contacting a caregiver to arrange immunisation. During the meeting it was determined who would be responsible for enacting the agreed action plan depending on the concerns identified.

### Evaluation

The following two evaluation methods were used to capture the learning:

- An electronic survey (using SurveyMonkey) was designed to capture feedback on the study process (Supplementary Figure S2);
- An anonymised detailed questionnaire was completed about three of the GP Connect children discussed per practice (Supplementary Figure S3).

The evaluation was designed to address the following questions:

- Do children who are late for immunisations have other family or social issues that may negatively impact on their wellbeing?
- Did this project change GP action plans or referral patterns for children?
- Process: How many GP and HV meetings were there and how long did they last; how easy were they to arrange; how useful were the meetings and the template; and how was the list of children to discuss generated?
- Communication issues: has the project changed the GP—HV relationship in any way?
- Outcomes: which referrals were made and did families attend? Were immunisations performed? Were other social or family issues identified? Were further actions needed and were the children subsequently discussed at a further GP and HV meeting?

Advice regarding information governance was obtained from the information governance team at NHS Lambeth Clinical Commissioning Group to ensure the pilot was compliant with the Data Protection Act. A data protection impact assessment (DPIA) questionnaire was submitted to the NHS North East London (NEL) Commissioning Support Unit.

## Results

Seven GP practices elected to participate in the project. Each practice was paid £1000 as compensation for participation. Most practices held four multidisciplinary meetings during the 6-month pilot, and 158 children in total were discussed across the seven practices (Table 1).

**Table 1.** Summary of GP Connect practices

Practice	Met regularly with HV in 2017 (before GP Connect)?	Number of registered patients (on day search was run)	Number of LEAP children aged 0–**3 years**	Number of individual immunisations that were late (in LEAP children)*	Total number of GP and HV meetings during pilot	Number of LEAP children discussed during pilot
1	No	12 602	135	181	4	20
2	Yes	6204	33	24	4	31
3	Yes	7786	174	270	4	20
4	No	7868	129	115	6	31
5	Yes	7311	85	21	2	10
6	No	12 055	411	578	4	26
7	Yes	Not provided	129	246	4	20
					**Total**	**158**

LEAP = Lambeth Early Action Partnership.

*Computer searches of children late for immunisations on the GP system are done by searching for each individual vaccine which is overdue and then combining the lists, meaning that children who are late for several immunisations appeared on several lists and so would be counted more than once in these numbers.

Nineteen practitioners — 10 GPs, two HVs, and seven other practitioners — involved in the project completed the electronic survey at the end of the pilot phase. All seven GP practices were represented. Detailed questionnaires were completed for 21 children (three per practice).

### Process

Most responders (13 of 17, 76%) indicated that it took <45 minutes to discuss five children and complete a template for each child. All responders (16 of 16) spent additional administrative time reviewing children’s patient notes and writing up action plans (Supplementary Table S1).

Nearly all responders (*n* = 15/16, 94%) found the multidisciplinary meetings useful. Most responders (*n* = 14/16, 88%) said that searching for children with late immunisations was helpful in identifying families with other issues that can negatively affect a child’s wellbeing. Three-quarters of responders (*n* = 9/12, 75%) found the bespoke template a ‘useful’ or ‘somewhat useful’ tool for stimulating discussion between GPs and HV; three (25%) did not. Seven out of 15 responders (47%) said that the GP–HV relationship was improved because of the pilot, and eight (53%) said the relationship remained unchanged. Table 1 contains information about the GP—HV relationship before the GP Connect project.

Responders were able to provide free-text feedback as well:

*'I know who the HV is now, and more about how they work. I hope it is a regular meeting eg, monthly.'* (GP, practice 5)

*'We are now seeing the health visitors again and feel we can bypass SPA* [single point of access referral to health visitors] *in certain circumstances, which enhances the relationship from my end.*' (GP, practice 1)

Seven out of 15 responders (47%) said that the pilot changed their action plans or referral patterns in some way; two (13%) said they changed very little; and six (40%) reported no change (Supplementary Table S1).

More than half of responders (8 of 13, 62%) indicated that participating in the pilot changed the way they thought about providing care to children aged 0–3 years and their families. The need to understand the complexities of a child’s home environment, and for effective interdisciplinary working and improved continuity of care, were highlighted as important factors in providing better care (Supplementary Table S1).

### Practice: care planning and provision

Detailed questionnaires provided more in-depth information about the different types of issues identified and the care plans for 21 children. Other issues of importance were identified in 57% (12 of 21) of children’s cases. Some of these were newly identified issues, including one new ‘child in need’ code entered because a sibling was on the child protection register, mental capacity concerns in a parent, or homelessness. The different issues identified are summarised in Table 2.

**Table 2.** Questionnaire results

Detailed questionnaire results: ACEs or other risk factors (developmental, medical, social) identified and recorded for 21 children (three children per practice)	Number of children (out of 21)
Frequent attender A&E (>2 times in 12 months)	8
Calls to out-of-hours GP	1
Split family (children divided or living with another carer)	2
Did not attend developmental reviews	1
Mother thought child was registered at a different practice	1
HV 1-year review: child not crawling	1
HV unable to contact family	1
Child very active, HV to assess and refer to GP if felt to be a medical issue	1
Maternal concerns re: speech and language development	1
Maternal mental health problems; history of mental health illness; mother low mood postnatally	3
Homelessness	1
Previous domestic abuse	1
No recourse to public funds	1
Mother does not speak English	1
Poor family relationship	1
Social isolation	2
Not engaging with services or difficulty engaging	2
Ongoing social services contact with family	4
Ongoing follow-up by GP or other specialists	6
Documented evidence that parents previously refused immunisation	5

ACE = adverse childhood experiences. HV = health visitor.

Table 3 summarises the different care plans enacted. Advice to carers about being late for immunisation was given in 90% (*n* = 19/21) of cases. Thirty-eight per cent (*n* = 8/21) of children had documented evidence of subsequently catching up on immunisations. A common outcome was ‘referral to HV’, which happened in 76% of cases (*n* = 16/21). ‘Referral to GP’ happened in 19% of cases (*n* = 4/21).

**Table 3.** Care plans

Action	Number of children (out of 21)	Attendance documented
Referral to children’s centre	1 (5%)	0 (0%)
Referral to health visitor	16 (76%)	9 (56%)
Referral to community paediatrics	1 (5%)	1 (100%)
Referral to GP	4 (19%)	4 (100%)
Advice to caregiver about immunisations	19 (90%)	N/A
Any other issues identified	12 (57%)	N/A
Other action points	5 (24%)	N/A
Child discussed at further HV and GP meeting	7 (33%)	N/A
Evidence child had immunisation	8 (38%)	N/A

HV = health visitor.

Of those families referred to HV, 56% (*n* = 9/16) were contacted successfully for further discussion. Of the seven that were not contacted, one family was uncontactable despite multiple attempts, and one family had a future date scheduled. In two cases the HV decided not to contact the family as there were clearly documented recent discussions and families had decided to not vaccinate their children. One GP said they were unable to access the HV notes to check whether the family had been contacted by the HV. This may have been lack of awareness of how to do this or owing to IT issues.

## Discussion

### Summary

GP Connect represents an innovative and sustainable approach to proactively identifying children potentially at risk of poor outcomes by using existing resources: GPs, HVs, computer records, and routine multidisciplinary meetings. This study illustrates that it is possible to use one indicator of risk (‘late for immunisations’) to identify children who may benefit from interdisciplinary sharing of information. This methodology resulted in several favourable outcomes; for example, more than one-third of children who were discussed caught up on their immunisations, and families with other issues that may negatively affect a child’s wellbeing were identified. Resources were then more effectively targeted to these families; for example, early help with social services support, local children’s centre services, HV, GP, or other primary care practitioner support.

### Strengths and limitations

This study used existing resources already in place, and although it involved a limited number of practices, clinicians, and children, it utilised a practical and readily adaptable approach and can easily be scaled up for wider study and/or implementation.

Costings were not directly broken down, apart from £1000 paid to practices for estimated administration and clinician time. This would be an important area to further quantify so resources can be adequately allocated.

It must be noted that some children who are up to date with their immunisations will also have ACEs or other markers of poor outcomes, therefore ‘late for immunisations’ should not be used in isolation to identify these children, but may be a useful additional risk marker. Other possible proxy markers, such as repeated A&E attendances and missed outpatient appointments, may also be useful; however, variations in practice coding of these events can limit their usefulness.

There were relatively small numbers of responders to the survey, and although all practices were represented, there were few HV responses so their views may be under-represented. Not all questions in the electronic survey were answered by all participants, which can affect interpretation of results.

Further work looking at modification of the bespoke template to guide the HV and GP discussions is also needed. Possible reasons for not finding the template useful may include the following: feeling it is restrictive or burdensome; the GP practice and HV already being skilled in having discussions about families; or some responders not being the professional who completed the template. The pilot also showed equivocal results regarding whether the template changed health professionals' action plans.

While the findings do not reveal large numbers of families who should have been referred to social services, it was discovered that current involvement of social services with the family was not always coded for on the GP electronic system.

### Comparison with existing literature

Previous studies have suggested poor recording of ACEs in GP records.^11^ Only 0.4% of GP patients in Scotland had a record of any code mapped onto the ACE questionnaire, contrasting with self-reported rates of 47% in patients. This is the first study the authors are aware of using late for immunisations as a proxy indicator for identifying children at risk of poor outcomes or those who may have suffered ACEs, thus enabling GP practices to have a practical and easy method for identifying these families.

### Implications for research and practice

This study suggests that searching for children who are late for immunisations may help identify families with potential risk factors for poor outcomes. These families can then be supported by existing early years services so resources can be targeted to those who need them most.

Communication between professionals was a key theme in this study. Before the pilot, some GP practices had regular meetings and a good relationship with their HV. However, some practices did not, and saw the pilot as a way to encourage a more collaborative relationship. One clear benefit of the pilot was the information sharing between GPs and HVs, which enabled a more detailed family picture to be recorded in GP notes. Responders mentioned the need to understand the complexities of a child’s home environment and the need for effective interdisciplinary working to improve continuity of care.

It would be useful for GPs to have automatic access to a family’s HV classification (community, universal, targeted, specialist)^12^ via joint computer systems. Allowing information to flow between different IT systems will be essential in the future as most data is saved electronically. The London Care Record,^13^ which wasn’t in place at the time this study was held, should facilitate this exchange of information.

A further larger qualitative study exploring the GP–HV relationship, and interdisciplinary working to improve child outcomes, could also be considered; this could be linked into a catch-up programme for immunisation. This study suggests that simply sending out reminder letters to families whose children are late for vaccinations is unlikely to work. Instead, families may need more personalised support with often multiple socially complex circumstances to enable them to have their children fully vaccinated.

This pilot used existing services that enables it to be sustainable if adopted more widely. Interdisciplinary working via GP and HV meetings should be considered essential practice and allowing time and allocating resources to maximise the usefulness of these meetings should be considered. Since the start of the COVID-19 pandemic, these meetings have been held virtually, and it will be useful to explore if this method is more acceptable, sustainable, and easier to arrange than face-to-face meetings.

## Data

The dataset relied on in this article is available from the corresponding author on reasonable request.
